# Comparing Mobility-Based PM_2.5_ Concentrations and Activity Satisfaction in Beijing between 2012 and 2017

**DOI:** 10.3390/ijerph20021386

**Published:** 2023-01-12

**Authors:** Jingwen Rao, Jing Ma, Yanwei Chai

**Affiliations:** 1 Beijing Key Laboratory for Remote Sensing of Environment and Digital Cities, Faculty of Geographical Science, Beijing Normal University, Beijing 100875, China; 2 College of Urban and Environmental Sciences, Peking University, Beijing 100871, China

**Keywords:** air pollution, subjective wellbeing, mobility, portable sensor, China

## Abstract

Although the negative effect of air pollution on life satisfaction has been examined in many studies, comparative analyses of mobility-based exposures to air pollution and momentary wellbeing have been rare to date, despite the fact that they are essential to improve wellbeing. Drawing on individuals’ space–time trajectories of two surveys conducted in 2012 and 2017 in Beijing, we investigate the temporal variations in activity satisfaction and mobility-based air pollution from monitoring stations and real-time air pollutant sensors, respectively. Furthermore, we explore how mobility-based air pollution dynamically influences activity satisfaction. The results show that air quality in Beijing improved from 2012 to 2017, and activity satisfaction increased as well. The negative relationship between them is more significant on workdays but insignificant on weekends. Moreover, real-time air pollution data show higher accuracy than monitor-based data, which suggests that future studies should pay more attention to real-time air pollution assessments.

## 1. Introduction

Subjective wellbeing (SWB) can be defined as how a person evaluates his or her life in both cognitive and affective ways, which contains both life satisfaction for the long term and momentary wellbeing, such as activity satisfaction and travel satisfaction [1,2]. As it is a crucial variable to evaluate the development of individuals or regions, many psychologists and economists have devoted much attention since the early 20th century [3]. In the 1970s, Richard Easterlin and other researchers observed the “Easterlin paradox”, showing no corresponding increase in SWB as income grew substantially [4]. Later, they considered China an excellent example because satisfaction data from the World Value Survey (WVS) indicated that with economic growth, Chinese citizens’ life satisfaction did not increase until 2005, showing a U-shaped trend [5,6]. Some researchers refuted that based on the Chinese General Social Survey (CGSS), saying Chinese people’s happiness has increased rather than decreased since 2003, which might be caused by various sample capacities and wellbeing scales [7,8]. These studies mainly focused on the increase or decrease in life satisfaction, which measures wellbeing in the long run. However, momentary wellbeing is also important [9,10], whereas temporal variations in momentary wellbeing, such as activity satisfaction, have received scarce interest to date.

Whether the WVS indicated a decrease in life satisfaction or the CGSS data suggested a minute increase in wellbeing, SWB could not be explained by rapid economic growth only [8,11]. Instead, to enhance the quality of life, the public and the government have paid more attention to the environment, especially air quality. In the past two decades, many scholars have investigated the negative relationship between air pollution and SWB, which differed across various regions [12,13,14]. However, there were still two limitations to these studies. First, almost all studies conducted sampling surveys about long-term life satisfaction on the macroscopic scales of countries [14], regions [15], cities, or communities [12,13]. Little attention has been paid to how air pollution affects our daily activities and the corresponding activity satisfaction, which have cumulative effects on long-term wellbeing and are important parts of SWB [9,10]. Second, by taking the static place of individuals’ residences as the sole reference geographical coordinate, most studies acquired air pollution data from monitoring stations. This approach was limited because convenient transportation gave people more mobility, and they usually went outside their residences, such as when conducting work activities [16]. The mismatching of air pollution and mobility might result in biases and inaccuracies in the analysis [17].

This is the case, particularly in developing countries such as China, where air pollution is serious and varies greatly in space and time. With the rapid development of the economy and urbanization, Beijing has suffered severe air pollution for a long time, which has posed a major threat to the health and wellbeing of its citizens. In the autumn and winter of 2012, the frequent heavy air pollution episodes in Beijing and the surrounding region attracted much attention from the media and the public. Hence, the China Meteorological Administration regarded air quality as a national interest in 2013 [18]. In September 2013, after the State Council issued the Air Pollution Prevention and Control Action Plan, the Beijing government also published the Beijing Clean Air Action Plan 2013–2017. Based on the comprehensive implementation measures from legal, economic, technical, and administrative aspects, the 5-year plan launched eight pollution reduction projects, including coal to gas, vehicle emission control, fugitive dust mitigation, and green coverage improvement. After 5 years of continuous efforts, the obvious decrease in annual average PM_2.5_ concentrations in Beijing from 89.5 µg/m^3^ in 2013 to 58 µg/m^3^ in 2017 indicated that the action plan achieved remarkable effects [19]. However, how mobility-based air pollution dynamically influences individuals’ daily activity satisfaction has rarely been investigated to date. Therefore, it is significant to carry out a comparative study of air pollution and momentary wellbeing in the context of clean air action plans.

In this study, we aim to explore the dynamic variations in mobility-based air pollution exposures and momentary wellbeing in the case of several communities in the Shangdi-Qinghe area, Haidian District, Beijing. Based on the data from two surveys conducted in 2012 and 2017, we first compare the variations in activity satisfaction across the 2 years. We then estimate the mobility-based PM_2.5_ concentrations from hourly monitoring stations and real-time portable air pollutant sensors and compare air pollution exposure across different years and assessments. Furthermore, we build a series of binary logistic regression models to investigate how activity attributes, socioeconomic variables, air pollution, and year are associated with activity satisfaction, how the two measurements of PM_2.5_ concentrations make a difference in modeling results, and how these variables interact with two different years to influence momentary wellbeing. Overall, this paper provides a scarce comparative analysis of activity satisfaction and mobility-based air pollution and makes timely contributions to better understand the dynamics of momentary wellbeing in urban China.

## 2. Methodology

### 2.1. Activity-Travel Survey of Beijing Residents in 2012

**Figure 1:**
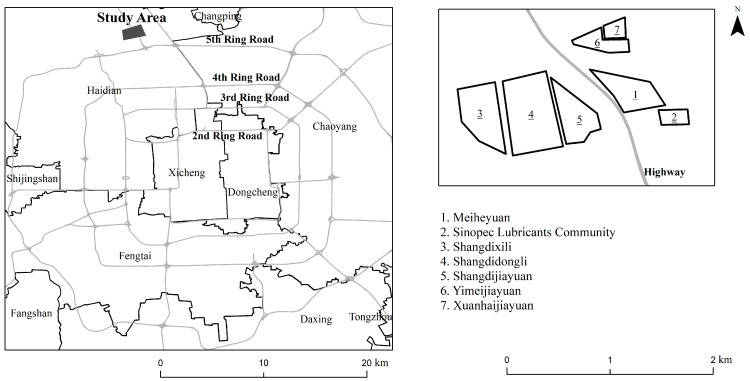
Study area.

As shown in Figure 1, the activity-travel survey of Beijing residents was conducted in the Shangdi-Qinghe area, which is one of the largest residential areas in Beijing and is located adjacent to the fifth ring road in the north of Haidian District. This survey area is representative of typical diverse housing types, including affordable housing neighborhoods, price-restricted commodity housing, and work unit compounds. A multistage clustering sampling strategy was employed to enhance sampling representativeness, and 23 residential communities and 19 companies were selected in the study area, while a stratified sampling strategy was used in each community to select approximately 1.0% of the population in each unit. We conducted the survey from October to December 2012, and each respondent was required to enroll in it for a week. In total, 791 respondents participated in the project, and 709 participants completed the questionnaires with valid data, including 480 residents and 229 employees.

The survey content is composed of three parts, including the main questionnaire, GPS trajectories, and activity diary. We asked each respondent to fill in the main questionnaire on the web to record their household and socioeconomic attributes. Each participant was also required to carry Garmin GPS 60CSx handheld receivers to record their daily mobility trajectories during the week. At the end of each day, participants were asked to complete the activity diary in the web diary application, wherein they could review their GPS trajectories. The day reconstruction method (DRM), where the diary was designed, and the GPS trajectories helped reduce recall biases and acquire more accurate information [20]. Based on the diaries, we collected detailed information for each activity conducted during the day, such as the starting and ending times, activity types, companionship, and degree of satisfaction with each activity.

### 2.2. Daily Activities and Environmental Health Survey of Beijing Residents in 2017

In 2017, we conducted a daily activities and environmental health survey in the Meiheyuan community, which is part of the Shangdi-Qinghe area and is also representative of all three housing types. We adopted a stratified sampling procedure and randomly selected participants, accounting for approximately 2.0% of the population, based on their apartment numbers. The survey was carried out from December 2017 to February 2018, with each respondent participating over a 2-day period containing a continuous workday and a weekend. In total, 117 participants were enrolled in the survey via six waves, with 112 residents providing valid data.

Based on the three parts of the survey in 2012, the 2017 project consisted of four parts, with real-time air pollution added. Each participant was asked to carry a small shoulder bag, which contained a GPS-equipped smartphone and a portable air pollutant sensor, to collect the mobility trajectories and real-time PM_2.5_ concentrations. The portable air sensor was the AirBeam from HabitatMap, which logged the real-time PM_2.5_ concentrations at one-second intervals. Moreover, investigators provided each respondent with their two-day GPS trajectories to help them fill in the DRM-based two-day activity diary on paper.

### 2.3. Data and Sample

To match the data in 2017, we filtered the data in 2012 based on participants, area, and time. First, we excluded 229 participants who worked for the 19 companies in the Shangdi-Qinghe area because only residents were investigated in 2017. Then, we selected the participants living in the Meiheyuan community in the 2012 survey and their surrounding communities to match the sample size in 2017 (as shown in Figure 1). Moreover, for each resident, we randomly selected Friday and Saturday or Sunday and Monday from a week-long survey in 2012 to correspond to the 2-day period survey containing a continuous workday and a weekend in 2017. Finally, after excluding the participants with null values and participants with <5 activity episodes, we obtained the final data in 2012, with 1301 activity episodes derived from 117 participants, while the 2017 final data had 1393 activities from 84 residents. Compared with the average of 17 activity episodes in 2017, the selected participants in 2012 reported 11 activity episodes on average.

Table 1 summarizes the sample characteristics of the socioeconomic attributes of participants and activity attributes in 2012 and 2017. The residents in 2012 resembled the participants in 2017 in terms of gender, although the local residents, employees, and the higher educated were overrepresented in 2012. This might be caused by the nuances of the study areas between 2012 and 2017. Regarding the activity attributes, the proportions of work activities and activity durations of less than 1 h in 2012 were less than those in 2017, possibly because of the different investigation methods. However, the differences between the two samples are not as important when we focus on the relationships between variables [9].

### 2.4. Measuring Mobility-Based Air Pollution Exposure

In this study, we estimated mobility-based air pollution exposure in two ways: hourly monitoring station assessment and real-time assessment at one-second intervals. After obtaining the hourly PM_2.5_ concentrations from 35 monitoring stations in Beijing during the two surveys provided by the Beijing Municipal Environmental Monitoring Centre (BMEMC), we used the inverse distance weight (IDW) to create 24 hourly PM_2.5_ concentration surfaces covering Beijing for each day during the two surveys. Based on the hourly PM_2.5_ concentration surfaces and participants’ mobility trajectories in space and time, we extracted the average PM_2.5_ concentrations for each activity episode, which surpassed the traditional static assessment of air pollution at residential locations [16]. Moreover, in the 2017 survey, portable air pollutant sensors were equipped to collect real-time PM_2.5_ concentrations at one-second intervals for each participant. Using the starting and ending times of each activity in the activity diary, we estimated the average real-time air pollution at the activity episode level for participants in 2017. In the subsequent analyses, the objective air pollution data were classified into five categories: <35 μg/m^3^ (1), 35–75 μg/m^3^ (2), 75–115 μg/m^3^ (3), 115–150 μg/m^3^ (4), and >150 μg/m^3^ (5), which takes into account the ambient air quality standard of the People’s Republic of China (GB3095-2012). For the two measurements of air pollution, two datasets were established. Dataset 1 included the PM_2.5_ concentrations estimated by 35 monitoring stations for the two surveys, while dataset 2 replaced the air pollution monitoring station data in the 2017 survey with the real-time data in 2017.

### 2.5. Analysis and Model

This study aims to compare the mobility-based PM_2.5_ concentration and activity satisfaction between 2012 and 2017. We first used all data to draw a line chart and a violin chart to analyze the variations in activity satisfaction and air pollution from 2012 to 2017. Then, we selected a weekday and a weekend in 2012 and 2017, respectively, to show the activity locations and air pollution exposure at four time points.

Logistic regression models were employed in this research to examine the relationships between these explanatory variables and momentary wellbeing. The outcome variable of this study was activity satisfaction, which was measured at the five-point level by using the Likert scale, ranging from 1 (very dissatisfied) to 5 (very satisfied). Based on the distribution of momentary wellbeing and prior studies [21,22], we dichotomized activity satisfaction: 0 for very dissatisfied, dissatisfied, and fair and 1 for the remaining two categories representing satisfied. In addition to air pollution and year, variables about individual socioeconomic conditions and activity episodes were selected as explanatory variables, which were proven to be related to wellbeing in previous studies [9,22,23]. The final activity satisfaction equation is as follows:

(1) $$\log\left(\frac{p}{1-p}\right)=\alpha +\beta A+\gamma S+\delta P+\phi Y$$

where *p* represents whether the respondent is satisfied with the activity, *A* refers to activity attributes (e.g., activity type, duration, and workday), *S* includes the five socioeconomic variables, *P* represents the measured air pollution exposure in five categories, and *Y* refers to year (1 for 2017 and 0 for 2012). *β*, *γ*, *δ*, *ϕ* are regression coefficients, and *α* is the regression intercept, which both need to be estimated. After checking potential collinearity using Pearson correlation and standardizing for the two continuous variables, age, and duration, logistic models were built in SPSS software.

## 3. Descriptive Analysis

### 3.1. Comparing Activity Satisfaction across the Two Years

**Figure 2:**
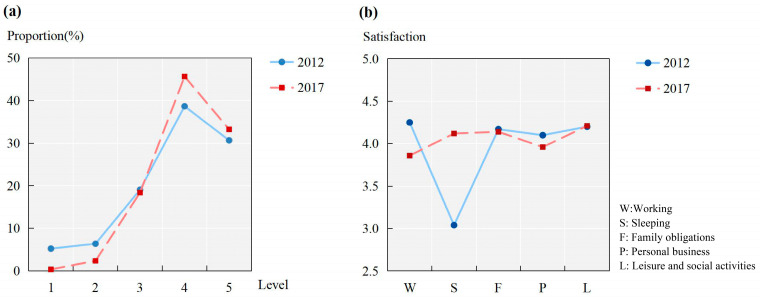
(**a**) Proportion of activity satisfaction and (**b**) average satisfaction level for different activity types.

As individuals’ activity satisfaction changes over time, we compared the activity satisfaction proportions in five levels between 2012 and 2017. As shown in Figure 2a, the distribution of activity satisfaction in 2012 was similar to that in 2017. For instance, approximately 19% of activities were evaluated as moderate, with more than 65% of activities reported as satisfied or very satisfied. The activity satisfaction at very unsatisfactory and unsatisfactory levels accounted for 5.23% and 6.38% of the samples in 2012, respectively, which decreased to 0.38% and 2.37% in 2017. In 2012, the satisfaction proportion reached the highest satisfaction level with a proportion of 38.66%, and the very satisfied level was the second highest with a proportion of 30.67%. In contrast, 45.66% and 33.27% of activities were evaluated as satisfactory and very satisfactory in 2017. These results indicate that there might be an increase in individuals’ activity satisfaction from 2012 to 2017.

Moreover, we estimated the average satisfaction in five activity types and compared it across the two years. As shown in Figure 2b, the average satisfaction with sleeping in 2012 was the lowest, approximately 3.0, while all other four types in 2012 were higher than 4.0. This suggests that participants in 2012 might have suffered poor sleeping quality. Personal business was the second lowest, with a value of 4.10. Working in 2012 reported the highest average activity satisfaction, reaching 4.25, followed by leisure and social activities and family obligations at 4.20 and 4.17, respectively. However, this trend is very different from that in 2017. With respect to 2017, working was estimated to be the lowest at 3.86, while leisure and social activities were the highest at 4.21. For family obligations in 2017, the average satisfaction seems similar to 2012. Compared with 2012, the average satisfaction with sleeping had a great improvement, with a value of 4.12 in 2017. Personal business in 2017 still held the second lowest satisfaction at 3.96 and was less satisfactory than that in 2012.

### 3.2. Comparing Air Pollution across the Two Years

**Figure 3:**
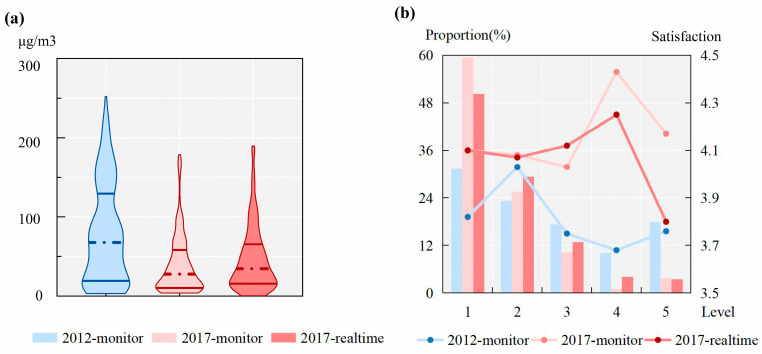
(**a**) Violin plot of objective air pollution and (**b**) average satisfaction at different pollution levels.

Figure 3a presents the distributions of three original air pollution datasets, including density (violin width), median (center dotted line), and two quartiles (two solid lines). As shown in Figure 3a, PM_2.5_ concentrations in the 2012 monitor dataset ranged from approximately 0 to 250 μg/m^3^, with the upper quartile up to 125 μg/m^3^. The extents in both the 2017 monitor and 2017 real-time datasets were below 200 μg/m^3^, with 75% of activities in 2017 exposed to PM_2.5_ concentrations below 75 μg/m^3^. Regarding data density, for the 2017 monitor and 2017 real-time datasets, after surpassing the medians, as PM_2.5_ concentrations increased, the probability density decreased rapidly. However, PM_2.5_ concentrations in 2012 were still widely distributed to high pollution extents. We then classified air pollution into five categories based on the Chinese national standard: <35 μg/m^3^ (1), 35–75 μg/m^3^ (2), 75–115 μg/m^3^ (3), 115–150 μg/m^3^ (4), and >150 μg/m^3^ (5), and then calculated the proportions in Figure 3b. The columns in Figure 3b show that for more than 75% of the activities in 2017, the exposures to air pollution were below 75 μg/m^3^, while only 54% of the activities in 2012 matched this level of pollution. Figure 3a,b both indicate that compared with 2017 participants, participants in 2012 were exposed to more serious air pollution, and air quality improved in 2017 due to the clean air action plan of the Beijing government.

Moreover, Figure 3a,b also shows slight differences between the 2017 monitor and 2017 real-time datasets. As shown in Figure 3a, the maximum, median, and two quartiles of the 2017 real-time dataset were slightly larger than those of the 2017 monitor, despite the similar appearances of the two violins. However, the 2017 real-time dataset had a lower minimum compared with the 2017 monitor. Regarding the data distribution, air pollution assessed by mobility-based monitoring stations was more concentrated in the lower quartile, while mobility-based real-time PM_2.5_ concentrations were distributed more widely. In addition, the differences in Figure 3b are more obvious. Approximately 60% of the activities were exposed to monitoring-station-assessed air pollution below 35 μg/m^3^, while this value was decreased to 50% in the real-time assessment. The proportion of exposures above 150 μg/m^3^ was 3.8% in the 2017 monitor dataset but 3.4% in the 2017 real-time dataset. Due to the differences in PM_2.5_ concentrations between outdoor and indoor environments, monitoring station assessments might overevaluate serious air pollution (Ma et al., 2020a). In contrast, real-time assessment increased the proportion of PM_2.5_ concentrations ranging from 35–150 μg/m^3^.

To better illustrate the connections between objective air pollution and daily activity satisfaction, we further drew the average activity satisfaction in each pollution category using three polylines in Figure 3b. As shown in this figure, average activity satisfaction in 2012 was evaluated at a relatively low value (below 3.8), with air pollution exposures above 75 μg/m^3^. As the air pollution in Beijing had improved in 2017 and residents were less sensitive to air pollution, such a trend does not exist in the 2017 monitor dataset. However, average activity satisfaction decreased rapidly and obviously when real-time air pollution surpassed 150 μg/m^3^. This indicates that real-time air pollution is more associated with activity satisfaction than monitoring station assessment.

### 3.3. Activity Distribution on Workdays and Weekends

**Figure 4:**
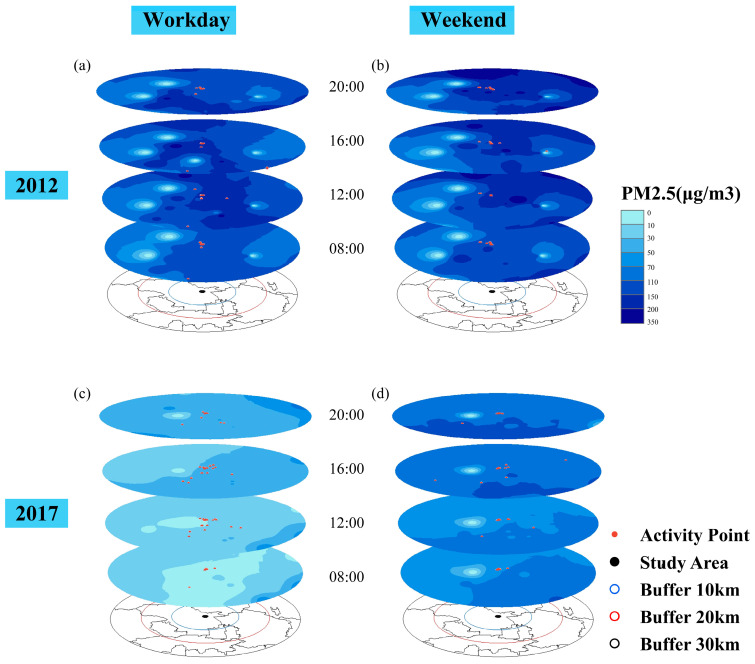
Real-time activity points and monitored air pollution at four times on (**a**) workdays in the 2012 dataset, (**b**) weekends in the 2012 dataset, (**c**) workdays in the 2017 dataset, and (**d**) weekends in the 2017 dataset.

To help illustrate the spatiotemporal dynamics of PM_2.5_ concentrations and human mobility, we chose four days, including a typical workday and a weekend day in 2012 and 2017, respectively, and four time points, equally spaced from 8:00 to 20:00, to interpolate the hourly air pollution layer based on the monitoring-station-assessed PM_2.5_ concentrations and record the respondents’ real-time activity points in 3D geovisualizations. In addition, each layer was clipped by a 30 km buffer of the study area, with more buffers shown in the base map. As shown in Figure 4, compared with 2012, the air pollution environment improved in 2017. Throughout the day, air pollution worsened over time. With respect to the activity distribution, it can be shown that there were fewer activity points in 2012 than in 2017. This may be because respondents in 2012 recorded fewer activity episodes over longer durations. Moreover, respondents had larger activity space on workdays in 2012, with some working beyond the neighborhood at distances of approximately 30 km. However, on weekends, activities were only carried out in a 10 km buffer. In contrast, respondents in 2017 chose workplaces within 20 km and had a larger activity space on weekends. This might be caused by improvements in human mobility and air pollution.

## 4. Modeling Results

**Figure 5:**
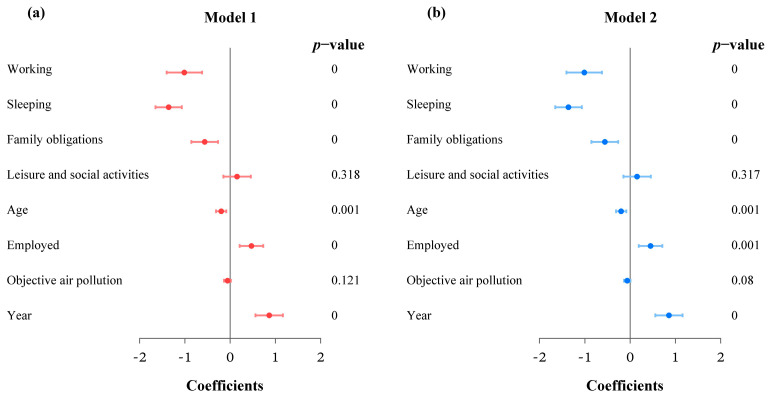
(**a**) Forest plot of some estimated coefficients in model 1 and (**b**) model 2.

Table 2 displays the estimated coefficients of the two logistics models in the different datasets, and Figure 5 draws the forest plots of some important coefficients in the two models. Both models include activity characteristics, socioeconomic variables, objective air pollution, and year. Model 1 uses the PM_2.5_ concentrations assessed by monitoring station data, while the air pollution in 2017 is replaced by real-time assessment in model 2. As shown in Table 2 and Figure 5, the estimation results of model 1 and model 2 are almost the same, except that the objective mobility-based air pollution is insignificant in model 1 but significant in model 2. Using real-time data, air pollution had a significantly negative association with activity satisfaction, possibly because real-time air pollution shows more accuracy than monitoring assessment. Moreover, year was significantly correlated with momentary wellbeing at the *p* = 0.05 level in both model 1 and model 2; that is, respondents in 2017 were more satisfied with their daily activities than those in 2012. For activity characteristics, only activity type was significantly associated with activity satisfaction at the *p* = 0.05 level, while duration and workday had no significance in momentary wellbeing. Among the five activity types, sleeping was significantly correlated with the least activity satisfaction, followed by working and family obligations, respectively, with leisure and social activities at the highest satisfaction level, though the difference was not significant. Regarding socioeconomic variables, age and employment showed significant associations with momentary wellbeing. Elderly respondents were more likely to be less satisfied with their daily activities than young people. Compared with unemployed individuals, employed individuals tended to have more enjoyable activities.

Despite the insignificant results of workdays in Table 2, the differences between workdays and weekends can be seen in the descriptive analysis. Therefore, we further divided workday and weekend samples from the original two datasets and then estimated the four models, which are shown in Table 3. Among all variables, only mobility-based air pollution made a difference in activity satisfaction between workdays and weekends. For activities on weekends, the negative association of air pollution with activity satisfaction was insignificant using both monitor data and real-time data. However, it was significant at the *p* = 0.05 level using real-time data when respondents conducted daily activities on workdays. This means that individuals might be more sensitive to air pollution on workdays than on weekends.

**Figure 6:**
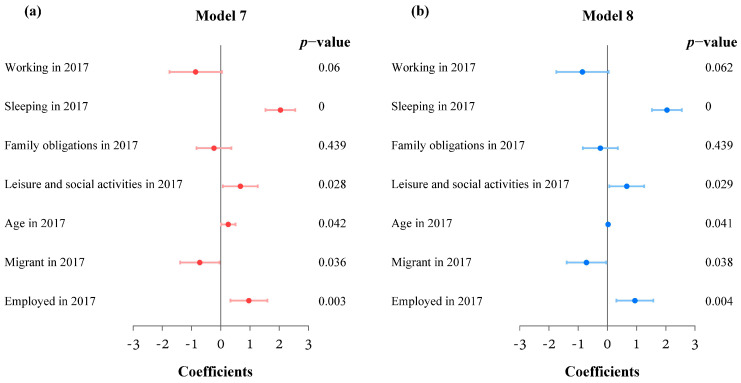
(**a**) Forest plot of some estimated coefficients in model 7 and (**b**) model 8.

To further explore the influences of some variables on satisfaction in different years, we performed multiplicative interaction analyses of year and other variables in model 7 and model 8. As shown in Table 4 and Figure 6, the estimated results were similar using monitor data and real-time data. There was no significant interaction between air pollution and year; however, interactions were significant for activity type and three socioeconomic variables. The satisfaction of work and study activities in 2017 decreased significantly at the *p* = 0.1 level, while the satisfaction levels of sleeping and leisure and social activities in 2017 significantly increased at the *p* = 0.05 level. Age and year had a significant positive interaction, which means that compared with 2012, the activity satisfaction of elderly people increased significantly in 2017. The interaction between employment and year was also positive and significant, while residence status interacted with year negatively and significantly. Migrants and unemployed respondents in 2017 were less satisfied with their daily activities than those in 2012.

## 5. Discussion and Conclusions

There are many studies on the relationships between air pollution and human wellbeing [12,13,14], as air pollution has attracted much attention worldwide, especially in developing countries such as China. Most of the studies used a set of cross-sectional data combined with air pollution data measured from monitoring stations to analyze how air pollution affects human happiness. However, comparative analyses of air pollution and activity satisfaction using both mobility-based monitoring-station-assessed and real-time-assessed PM_2.5_ exposure have been very deficient to date. In this research, we conducted a comparative analysis of air pollution and momentary wellbeing based on two surveys conducted in 2012 and 2017. First, we investigated the variations in activity satisfaction and mobility-based air pollution across the two years. Then, a quantitative analysis of logistic models revealed the effects of activity variables, socioeconomic attributes, objective air pollution, and year on daily activity satisfaction. Moreover, we compared the monitoring-assessed PM_2.5_ concentrations and the real-time measured PM_2.5_ concentrations in a series of different models and found that real-time air pollution had a more significant effect on momentary happiness. To the best of our knowledge, this is the first study to compare how mobility-based air pollution influences momentary activity satisfaction at a fine spatiotemporal resolution in different years.

As the results show, the air quality in Beijing improved from 2012 to 2017, which is closely related to the continuous efforts of the government. Objective air pollution has a significant negative impact on activity satisfaction, in line with previous studies [14,24]. However, further investigation indicated that this negative relationship became more significant on workdays but was insignificant on weekends, possibly because commuting and feeling work-related pressure might make people more vulnerable and sensitive to air pollution exposure. Moreover, our study also demonstrated higher accuracy and better model effect of real-time air pollution data than monitor-based data, which implies that the mobility-based microenvironment, especially the indoor microenvironment, is important but often ignored in prior studies.

Activity satisfaction increased from 2012 to 2017, probably due to an improvement in air pollution. The satisfaction levels of different groups also changed over the years. Compared with 2012, older people tended to reap more enjoyment from daily activities in 2017. The reason may lie in the reduction in economic pressure and the increasing attention of government and society to the old. Migrants in 2017 were likely to have lower satisfaction levels than those in 2012, which might be caused by the increasingly tightened population policy of Beijing. Additionally, employed respondents in 2017 contributed to higher wellbeing levels with their daily activities than those in 2012, possibly because the air pollution in Beijing was greatly improved.

There are some limitations to be acknowledged in this study. First, the survey in 2017 was not designed as a follow-up investigation to that in 2012, which led to some differences between the two survey samples. However, the two investigations were developed in similar study areas using the DRM, which offered the possibility for comparison. Future research can conduct follow-up investigations for the same respondents in the same area to conduct longitudinal analysis. Second, based on the first limitation, it is hard to deeply explore the causal relationship between air pollution variations and the increase in satisfaction during the period. Nonetheless, we analyzed the variations and compared the changes in PM_2.5_ concentrations and activity satisfaction across the two years at length, hoping to provide some reference value for future research. Third, the air pollution data from the monitoring station were outdoors, but people are expected to spend most of their time indoors, which leads to some bias in results by under- or overestimating air pollution exposure when conducting indoor activities [16]. However, this study also showed that the real-time air pollution data, which was collected by air pollutant sensors in a microenvironment, had better results than the monitoring station data. However, due to the lack of related equipment in the survey of 2012, we only collected these data in 2017, which limited our study. These kinds of data can be widely used in the future. Moreover, due to the limitation of surveys and data, we only compared two specific years. If conditions permit, future studies could compare more years.

## Figures and Tables

**Table 1:** Sample characteristics.

Variable	Description	2012 (%)	2017 (%)
Age	18–29	23.08	16.67
	30–39	50.43	26.19
	40–49	19.66	32.14
	>50	5.98	25.00
Gender	Male	46.15	46.43
	Female	53.85	53.57
Residence status	Migrants	9.40	20.24
	Local residents	90.60	79.76
Employment	Employed	92.31	72.62
	Unemployed	7.69	27.38
Education	College or above	84.62	65.48
	High school or below	15.38	34.52
Activity type	Working	3.69	10.55
	Sleeping	29.82	22.04
	Family obligations	12.61	17.37
	Personal business	33.97	28.79
	Leisure and social activities	19.91	21.25
Activity duration	≤1 h	40.05	46.31
	1~3 h	33.05	27.10
	3 h	26.90	26.45

**Table 2:** Estimated coefficients of logistics models using monitor and real-time data.

	Monitor Data	Real-Time Data
Variables	Model 1	Model 2
Duration	0.041	0.040
Workday	−0.013	−0.009
Type		
Working	−1.012 **	−1.013 **
Sleeping	−1.359 **	−1.360 **
Family obligations	−0.562 **	−0.561 **
Leisure and social activities	0.154	0.154
Age	−0.197 **	−0.199 **
Male	0.089	0.093
Migrant	−0.162	−0.155
Employed	0.470 **	0.451 **
Education	0.110	0.097
Objective air pollution	−0.059	−0.064 *
Year	0.862 **	0.857 **

Note: All estimates are standardized regression coefficients. * *p* < 0.1, ** *p* < 0.05.

**Table 3:** Estimated coefficients of logistics models by workday and weekend.

	Workday		Weekend	
	Monitor Data	Real-Time Data	Monitor Data	Real-Time Data
Variables	Model 3	Model 4	Model 5	Model 6
Duration	0.107	0.103	−0.014	−0.017
Type				
Working	−1.030 **	−1.031 **	−1.122 **	−1.126 **
Sleeping	−1.508 **	−1.510 **	−1.226 **	−1.225 **
Family obligations	−0.574 **	−0.571 **	−0.525 **	−0.523 **
Leisure and social activities	0.114	0.115	0.194	0.195
Age	−0.182 **	−0.192 **	−0.208 **	−0.204 **
Male	0.211	0.218	−0.016	−0.014
Migrant	−0.167	−0.156	−0.180	−0.182
Employed	0.558 **	0.529 **	0.383 **	0.371 **
Education	0.046	0.022	0.148	0.140
Objective air pollution	−0.109	−0.059 **	−0.033	−0.019
Year	0.722 **	0.862 **	0.984 **	0.994 **

Note: All estimates are standardized regression coefficients. ** *p* < 0.05.

**Table 4:** Estimated coefficients of logistics models for interactions.

	Monitor Data	Real-Time Data
Variables	Model 7	Model 8
Working in 2017	−0.861 *	−0.852 *
Sleeping in 2017	2.040 **	2.041 **
Family obligations in 2017	−0.236	−0.237
Leisure and social activities in 2017	0.670 **	0.665 **
Age in 2017	0.027 **	0.027 **
Male in 2017	−0.024	−0.019
Migrant in 2017	−0.722 **	−0.714 **
Employed in 2017	0.962 **	0.943 **
Education in 2017	0.182	0.174
Objective air pollution in 2017	−0.019	0.009

Note: All estimates are standardized regression coefficients. * *p* < 0.1, ** *p* < 0.05.

## Data Availability

Data available upon request to the corresponding author.
